# Effects of Alzheimer’s Disease on Visual Target Detection: A “Peripheral Bias”

**DOI:** 10.3389/fnagi.2016.00200

**Published:** 2016-08-17

**Authors:** Vanessa Vallejo, Dario Cazzoli, Luca Rampa, Giuseppe A. Zito, Flurin Feuerstein, Nicole Gruber, René M. Müri, Urs P. Mosimann, Tobias Nef

**Affiliations:** ^1^Gerontechnology and Rehabilitation Group, University of BernBern, Switzerland; ^2^ARTORG Center for Biomedical Engineering Research, University of BernBern, Switzerland; ^3^University Hospital of Old Age Psychiatry and Psychotherapy, University of BernBern, Switzerland; ^4^Perception and Eye Movement Laboratory, Department of Neurology and Clinical Research, University Hospital Inselspital, University of BernBern, Switzerland; ^5^Private Hospital WyssMünchenbuchsee, Switzerland

**Keywords:** visual exploration, Alzheimer’s disease, search strategy, target detection, eye movements, large hemispherical screen, visual attention

## Abstract

Visual exploration is an omnipresent activity in everyday life, and might represent an important determinant of visual attention deficits in patients with Alzheimer’s Disease (AD). The present study aimed at investigating visual search performance in AD patients, in particular target detection in the far periphery, in daily living scenes. Eighteen AD patients and 20 healthy controls participated in the study. They were asked to freely explore a hemispherical screen, covering ±90°, and to respond to targets presented at 10°, 30°, and 50° eccentricity, while their eye movements were recorded. Compared to healthy controls, AD patients recognized less targets appearing in the center. No difference was found in target detection in the periphery. This pattern was confirmed by the fixation distribution analysis. These results show a neglect for the central part of the visual field for AD patients and provide new insights by mean of a search task involving a larger field of view.

## Introduction

Alzheimer’s disease (AD) is the most common form of dementia, and refers to a progressive neurodegenerative disease, characterized by a decline in cognition, that influences the activities of daily living (Marshall et al., 2011). These activities rely on visual exploration, guiding our actions. For instance, during driving, the visual scene is constantly explored, in order to attend to relevant objects (Trick et al., 2004). Attending to relevant visual information in the extra-personal space relies on eye movements, an intact visual field, and on an appropriate level of performance for the action (Müri et al., 2005). Consequently, eye movement analysis, assessing saccades and fixations, has been often applied to reliably investigate visual exploration strategies (Zihl, 1995). Moreover, it has been shown that eye movements are also a valid physiological measure of the deployment of visual attention in space (Scinto et al., 1994). Other studies have also demonstrated that the cortical neural correlates of saccadic eye movements and visual attention shifting overlap, and that these two aspects are functionally related (Corbetta et al., 1998; Hoffman, 1998). Attention shifting and eye movements both rely upon a fronto-parietal network (Bisley, 2011) and, due to neurodegenerative processes in this network, impairments in the control of visuo-spatial attention may result (Cazzoli et al., 2015). In patients with AD, prominent deficits are present in the shifting of attention (Parasuraman and Haxby, 1993), in the inhibition of shifting to irrelevant but salient spatial locations (Danckert et al., 1998), in the shifting of the attentional focus between local and global features (Filoteo et al., 1992), and in the ability to select the focus of attention (Calderon et al., 2001).

This decline in the control of visual attention, also reflected in eye movement alterations, could be due to a less efficient visual search strategy when detecting targets. Indeed, several studies have demonstrated that visual processing is impaired in AD (Adlington et al., 2009; Bublak et al., 2011), and the pattern of eye movements of AD patients is less organized than the one of healthy controls (Lueck et al., 2000; Molitor et al., 2015). These differences in eye movement patterns have also been used for the validation of an assessment tool for dementia (Currie et al., 1991). Moreover, in the context of rehabilitation programs, neuro-visual trainings (e.g., saccadic and visual exploration trainings) have been applied in different populations of neurological patients (Kerkhoff, 2000). As a result of these rehabilitation programs, patients showed an overall amelioration of their saccadic eye movement patterns, with an increase of saccadic amplitudes, an increase of saccadic velocity, and a decrease of saccadic reaction times. Moreover, they could improve localization of fixations and saccadic eye movements, i.e., they could perform more precise saccades toward a given point, and could more easily perform saccades and pursuit eye movements in response to moving objects, as well as displaying a more organized search strategy. In order to develop a training with high ecological validity, a precise knowledge of the patterns of visual exploration of AD patients in a naturalistic environment is crucial.

In a study, where AD patients had to perform a simulated driving task and eye movements were recorded, Mapstone et al. (2001) showed that patients, as well as older healthy controls, focused their attention predominantly on the periphery, whereas younger healthy controls focused their attention predominantly on the central part of the visual scene. Analogously, Rösler et al. (2005) found that AD patients produced more peripheral fixations than healthy controls, and presented a deficit in disengaging attention from peripheral targets. These results have been obtained in experimental setups in which patients had to perform the task on a flat screen with search visual displays subtending a small visual angle. Critically, however, other studies have shown that wider visual angles and larger visual scene sizes can substantially influence the pattern of results, e.g., decreasing performances in healthy controls (Gibson, 2014), or increasing deficits in neurological patients with visuo-spatial attention impairments when attention has to be deployed over a large search array (Eglin et al., 1994).

An ecologically valid test of visual exploration is challenging to implement in laboratory conditions, especially when attempting to specifically assess peripheral target detection, which requires a setup with a large field of view. In the present study, we thus employed a setup composed of a hemispherical projection screen, which allows to present stimuli on a ±90° visual field. The aim of the present study was to better understand visual search performance, and the related visual search strategies, in AD patients compared to healthy subjects. In particular, we sought to apply a large visual angle for stimulus presentation, in order to assess the effects of the latter as a potential, crucial determinant of impaired visuo-spatial attention deployment during activities of daily living. To accomplish this goal, images of daily living were projected onto a hemispherical screen, and participants were free to move their eyes in order to locate targets within these images, while their eye movements were recorded.

## Materials and Methods

### Participants

Eighteen patients with AD, and 20 healthy controls with no indication of cognitive impairment, were recruited for the study. Patients eligible for the study were recruited from the Interdisciplinary Memory Clinic of the University Hospital of Old Age Psychiatry in Bern, Switzerland, and were all previously diagnosed with probable AD, according to the criteria laid down in the International Classification of Diseases, 10th Revision. Patients were assessed with the German version of the CERAD neuropsychological battery (Consortium to Establish a Registry for Alzheimer’s Disease; Morris et al., 1989). Additional clinical scales were also administered for the evaluation of the activities of daily living: the Bristol Activities of Daily Living Scale (Bucks et al., 1996), and the Functional Activities Questionnaire (Pfeffer et al., 1982). Finally, the Geriatric Depression Scale (Parmelee and Katz, 1990) was administrated to verify the presence and severity of depressive symptoms. Structural MRI data were also examined for all patients, in order to exclude any other brain anomaly.

The control group was recruited from the Seniors University of Bern, Switzerland. Healthy controls were assessed with the Montreal Cognitive Assessment (MoCA) screening tool (Nasreddine et al., 2005). The corresponding inclusion criterion for the control group was a cut-off score above 26 in the MoCA.

Exclusion criteria for both groups were color blindness, glaucoma, cataract, visuomotor disturbance, and insufficient or insufficiently corrected visual acuity.

The experiment was carried out in accordance with the latest version of the Declaration of Helsinki, and was approved by the local Ethics Committee. All participants gave written informed consent prior to participation to the study.

Demographics and clinical characteristics of the participants are summarized in **Table 1**.

**Table 1:** Demographics and clinical characteristics of the participants (Mean ± *SD*).

	Control subjects (*N* = 20)	AD patients (*N* = 18)	*P*-value
Age (years)	72.2 ± 3.4	74.3 ± 7.6	*P* > 0.05
Education (years)	12.2 ± 2.9	12.4 ± 3.1	*P* > 0.05
Gender (male:female)	10:10	8:10	*P* > 0.05
Global Cognition			
MoCA score	28.5 ± 1.1	19.4 ± 4.5	*P* < 0.001


*MoCA, Monreal cognitive assessment; Mean (±SD), *t*-test was performed to assess differences.*

### Experimental Setup and Procedure

**FIGURE 1:**
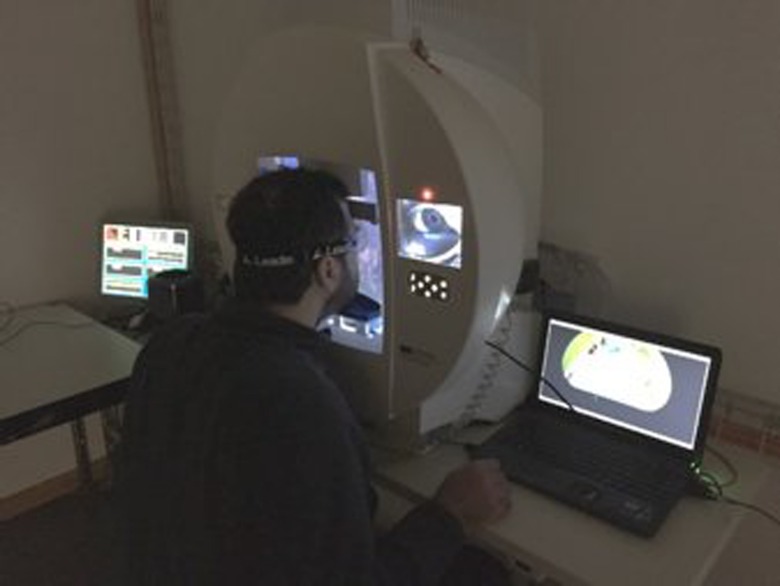
**Experimental setup during the experiment**.

The experiment consisted of a visual search task, where participants had to explore images presented on a hemispherical screen with a diameter of 60 cm (Octopus 900, Haag-Streit AG, Köniz, Switzerland). Participants were free to move their eyes, but were asked not to move their head, which was stabilized by means of a chin-and-head rest (**Figure 1**).

**FIGURE 2:**
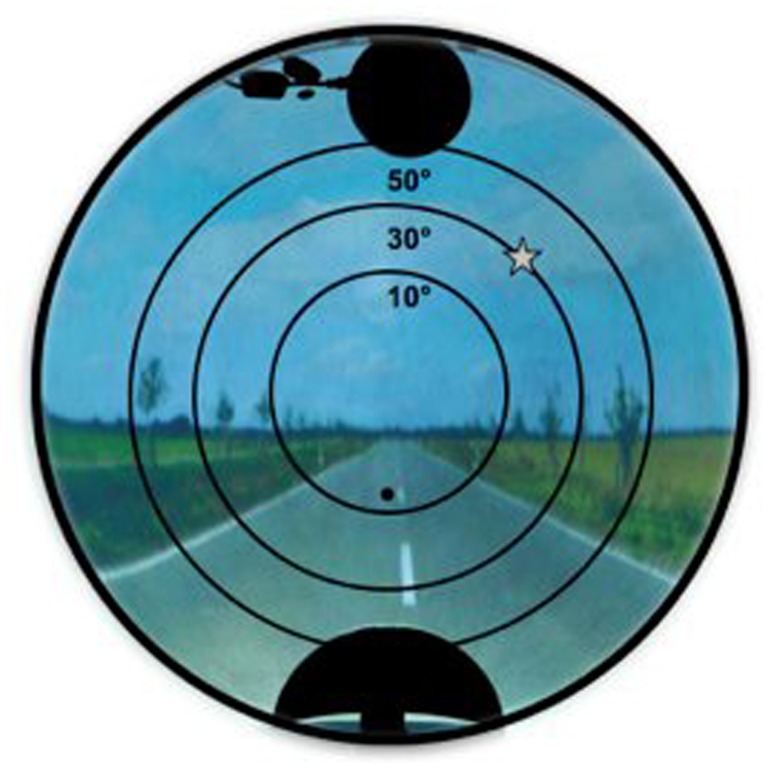
**Image projected on the hemispherical screen, with a target (star shape).** The grid of concentric circles, representing the three possible eccentricities for targets presentation, was not visible to the participants.

A custom developed mirror-projection system allowed to project images of everyday life (i.e., landscapes, streets, buildings, everyday objects) onto the hemispherical screen, covering ±90° field of view (Nef et al., 2014). Participants were instructed to press a response button whenever they located a target (a small gray star), and not to press the button when they located a distracter (a small gray triangle; of similar size, and of identical color and luminance with respect to the target shape). This design was thus similar to a classical Go–NoGo task. Targets and distracters were presented one at a time, at locations predetermined by a grid of concentric circles that was not visible to the participant (**Figure 2**). The grid determined the eccentricity of stimulus appearance, and comprised 36 possible positions. Every stimulus (target or distracter) remained on screen for 2 s. The inter-stimulus interval (ISI) was randomly varied between 0.5 and 2 s. Before the experiment proper, a practice run was carried out. The practice run was repeated as many times as needed, until the participants understood the task.

The responses to targets and distracters were recorded. The percentage of targets with a response (i.e., correct responses) for the eccentricities of 10°, 30°, and 50° was computed, as well as the mean reaction times to respond to targets at each of the three eccentricities. Moreover, the percentage of distracters with a response (i.e., incorrect responses) was computed for the eccentricities of 20° and 40°. By assigning distracters positions closer to the center and avoiding an overlap with targets positions, an enhanced distracter effect was intended without increasing the number of distracters (Honda, 2005). The positions of targets and distractors were based on the experimental set up by Gruber et al. (2014).

Eye movements were recorded by means of the integrated eye camera of the Octopus 900. The eye-tracking system was calibrated by means of a five-point-calibration procedure. Further details of the test setup are described in Gruber et al. (2014).

### Data Analysis

#### Target Detection and Eye Movement Analysis

The responses to targets and distracters, as well as the corresponding reaction times, were analyzed using MATLAB (MathWorks Inc.). Eye movement analysis was also performed using MATLAB. In a first step, the pupil position was recorded, using the five-point-calibration and an offline algorithm (Zito et al., 2014). In a second step, these positions were transferred to a polar space, separated into distance and angle, and mapped onto the hemispherical screen. For each participant, we calculated the percentage of fixations in four eccentricity areas (0–20°, 20–40°, 40–60°, and >60°), the mean fixation time, and the mean distance between gaze position and target position at the time point of target onset.

### Statistical Analyses

Statistical analyses were performed using SPSSv20 (IBM Corporation, Armonk, NY, USA). A mixed-model analysis of variance (ANOVA) was used for group comparison with the percentage of target recognition and reaction time measured at three eccentricities (10°, 30°, and 50°) and percentage of fixations measured at four eccentricity areas (0–20°, 20–40°, 40–60°, and >60°). A mixed-model ANOVA was also used to assess potential differences in the percentage of responses to the distracters, with the within-subjects factor eccentricity (20° and 40°) and the between-subjects factor group (patients, controls). *Post hoc* comparisons were performed by means of Bonferroni-corrected *t*-tests. Pearson’s r, the correlation coefficient served as effect size estimation of the group difference for target detection percentage at 10°, 30°, and 50°. A Greenhouse–Geisser correction was applied when the sphericity assumption was not met, as assessed by the Mauchly’s test of sphericity.

The comparisons between the two groups concerning the mean fixation time, and the mean distance between gaze position and target position at the time point of target onset, were carried out using independent-samples *t*-tests.

The significance level was set α < 0.05 for all analyses.

## Results

### Target Detection, Reaction Times, and Incorrect Responses to Distracters

**FIGURE 3:**
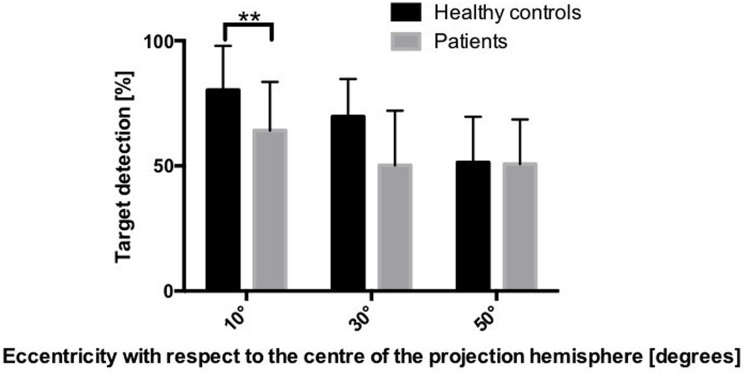
**Mean percentage of detected targets in the two groups, depending on the eccentricity.** ^∗∗^*p* < 0.01 (based on the Bonferroni correction for multiple comparisons).

**Figure 3** shows group differences in target detection. The mixed-model ANOVA did not show a main effect of group [*F*(1,36) = 1.740, *p* = 0.05], but revealed a main effect of eccentricity [*F*(2,72) = 57.61, *p* < 0.001], indicating diminished performance with greater eccentricity, irrespective of the group. Crucially, this main effect was qualified by a significant interaction between eccentricity and group [*F*(2,72) = 8.07, *p* = 0.001], indicating that the profile of responses across the different eccentricities was different for patients and controls. *Post hoc* pairwise comparison showed that the percentage of detected targets was significantly lower for AD patients than for healthy controls at 10° eccentricity (*p* = 0.024), but no significant differences were found between the two groups at 30° (*p* = 0.273) or 50° eccentricity (*p* = 1.434). Additionally, the effect sizes of these group differences showed a decrease with increasing eccentricity (for 10°: *r* = 0.427; for 30°: *r* = 0.277; for 50°: *r* = 0.118).

**FIGURE 4:**
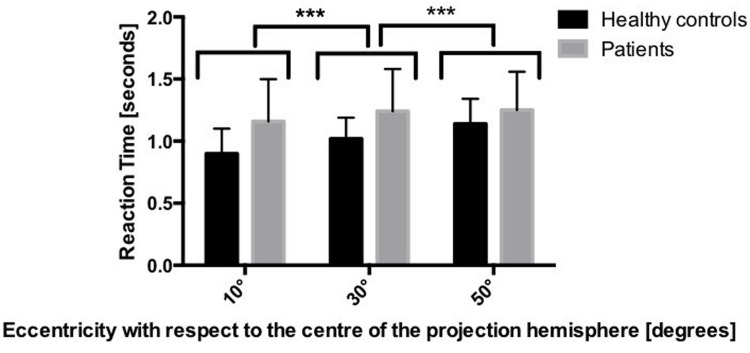
**Mean reaction times in the two groups, depending on the eccentricity.** ^∗∗∗^*p* < 0.001 based on mixed-model ANOVA.

The mixed-model ANOVA on the reaction times revealed a main effect of group [*F*(1,36) = 10.765, *p* < 0.05] showing that patients were slower than healthy controls, as well as a main effect of eccentricity [*F*(2,72) = 30.95, *p* < 0.001], indicating slower reaction times at larger eccentricities. This effect was not qualified by an interaction between eccentricity and group [*F*(2,72) = 2.11, *p* > 0.05], indicating that this pattern was equally present in both groups (**Figure 4**).

Regarding the incorrect responses to the distracters, the mixed-model ANOVA did not yield any significant main effect of group [*F*(1,36) = 2.264, *p* > 0.05) or of eccentricity [*F*(1,36) = 2.32, *p* > 0.05], nor any interaction between eccentricity and group [*F*(1,36) = 0.019, *p* > 0.05]. Hence, the percentage of incorrect responses to distracters was equivalent in healthy controls and AD patients, and was not influenced by eccentricity.

### Gaze Position

**FIGURE 5:**
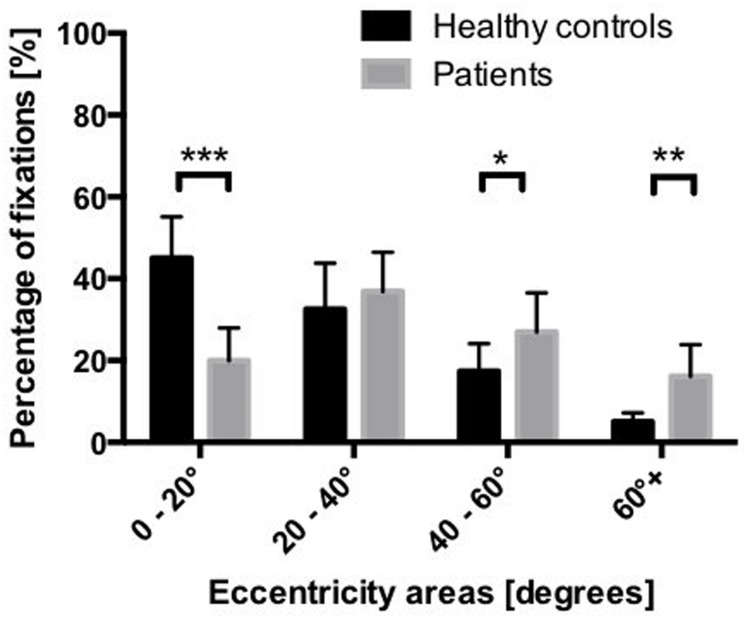
**Mean percentage of fixations in the four eccentricity areas of the hemisphere.** ^∗^*p* < 0.05, ^∗∗^*p* < 0.01, ^∗∗∗^*p* < 0.001 (based on the Bonferroni correction for multiple comparisons).

**Figure 5** depicts the percentage of fixations within the four eccentricity areas, for the two groups of participants. The mixed-model ANOVA revealed a significant main effect of eccentricity [*F*(3,34) = 27.806, *p* < 0.001], indicating lower fixation percentages at greater eccentricities, irrespective of the group. Crucially, this main effect was qualified by a significant interaction between eccentricity and group [*F*(3,34) = 6.426, *p* < 0.001], indicating that the pattern of fixations across the different areas of eccentricity was different for patients and controls. *Post hoc* pairwise comparisons revealed that patients produced significantly less fixations than healthy controls in the 0–20° eccentricity area, whereas they produced significantly more fixations than healthy controls in the 40–60° and in the >60° eccentricity areas.

No significant differences between AD patients and healthy controls were found regarding the mean fixation time [Healthy controls: Mean (in seconds) = 0.42; *SD* = 0.26; AD Patients: Mean (in seconds) = 0.39; *SD* = 0.32; *t*(36) = 1.207, *p* > 0.05].

Concerning the mean distance between gaze position and target position at the time point of target onset, healthy controls showed a significantly shorter distance than AD patients (Healthy controls: Mean (in angle) = 43.79; *SD* = 34.05; Patients: Mean (in angle) = 57.03; *SD* = 38.49; *t*(36) = -4.544; *p* < 0.001).

## Discussion

The aim of the present study was to examine the target detection performance and the visual exploration behavior in AD patients compared to healthy subjects, using a visual search task on a hemispherical screen, covering an extended ±90° field of view. The results showed that AD patients detected less target than healthy controls in central positions, but no differences were found between the two groups at eccentricities of 30° and greater. This is consistent with previous results, showing that AD patients have decreased performance, as compared to healthy controls, in target detection in the central part of the field of view (Rösler et al., 2000). When eccentricity increased, the results indicated a diminished performance in healthy controls, confirming the findings of a recent study (Gruber et al., 2014). However, this pattern of performance was not present in AD patients. Peripheral cues attract attention automatically, and cause a faster allocation of attention, while central cues need voluntary attentional control, and require greater processing time to be interpreted (Müller and Rabbitt, 1989). According to Jonides (1981), the voluntary attentional control and the automatic reflexive orienting differ in their automaticity and processing resources. While voluntary orienting in response to central cues relies on selective attention and needs additional processing resources, automatic orienting to peripheral cues is driven by perceptual identification (Molenberghs et al., 2009). Additionally, because of the natural tendency to respond toward the source of stimulation when making keypress responses, Molenberghs et al. (2009) suggested that peripheral cues automatically activate corresponding responses to stimulus, whereas central cues still activate the voluntary attentional control. This interpretation is supported by findings concerning neuroanatomical correlates. In fact, responses to peripheral, automatic cues are subtended by the superior colliculus (Crick, 1984), whereas voluntary attentional responses to central cues presumably involve the inferotemporal cortex and the posterior parietal cortex (Serences and Yantis, 2006). This might explain the differences between the two groups in target detection performance, since the superior colliculus remains relatively spared until the very severe stages of the disease, while temporo-parietal areas are already affected from early-stage AD (Jobst et al., 1994; Perry and Hodges, 1999; Montez et al., 2009).

Alzheimer’s disease patients needed significantly more time to detect targets and detected significantly less targets at 10° eccentricity. Thus, while healthy older individuals seem able to detect a target through peripheral vision, and immediately shift their attention upon it to process the available information, AD patients seem to have to explore for a longer period of time, until they attend to a point in space that is close to the target, and can finally detect the target, too. This hypothesis is in line with previous findings, showing that AD patients produce an increased number of fixations before fixating a given region of interest (Mosimann et al., 2004). This also relates to a higher response threshold, restricting covert attention shifting, and hampering the preparation of saccadic eye movements toward a target and, therefore, reducing search efficiency. In daily life, such a deficit put AD patients at risk to miss relevant information, resulting in inadequate reactions to the surrounding environment, such as, for instance, difficulties in finding items in a supermarket, using public transportation, or safely crossing a street.

Another explanation for the decreased target detection performance and the slower reaction times in AD patients relies in the impaired ability to move the eyes precisely and fast enough to explore the visual field. Although no significant differences were found concerning the mean fixation time, this explanation is still supported by the significant difference in distance at target onset between AD patients and healthy controls. Moreover, a larger distance to reach the next target onset could indicate that AD patients tend to focus their attention more on the periphery. In fact, the distance to reach the next target located in a peripheral area is larger if the participant’s attention is already focused on an opposite peripheral area. Conversely, when the participant’s attention is focused on a central area, the distance to reach the next target located in the periphery would be smaller. Indeed, behavioral results showed that AD patients have a different visual search strategy, focusing their search more toward the periphery, whereas healthy older controls focus their search more toward the center of the field of view. These results are partially in accordance with previous studies (Schlotterer et al., 1984; Mapstone et al., 2001), assessing the spatial allocation of visual attention in AD patients and in healthy controls. However, the present study, using a larger field of view, reveals a different pattern of results: what was considered as periphery in the previous studies, corresponds to paracentral areas in the present study. When patients have the possibility to explore a broad field of view, the center is neglected, creating a “peripheral bias”. These findings are relevant for a considerable amount of activities of daily living, in which a large visual field has to be taken into account, such as, e.g., during driving. This might also influence the way in which the abilities of AD patients are actually assessed. For instance, several current assessment methods are based on cognitive tasks on computer screens subtending limited visual angles. Such approaches would thus not reflect the biases described in the present report, which are observable under conditions with large visual angles.

Concerning incorrect responses to the distracters, no group differences were found. This is in line with the extant literature, suggesting that AD patients are not impaired in Go/No Go tasks until a certain disease severity stage (Zhang et al., 2007). However, this may apply only to distracters that differ from the target in terms of a single feature, as it was the case in the present study. In fact, it has been shown that in feature conjunction search (i.e., including distracters that are similar to the target with respect to more than one feature), AD patients have impaired response inhibition (McLaughlin et al., 2010).

Importantly, the current study provides new insights by means of a search task entailing a larger field of view. This allowed to show that, when AD patients are free to move their eyes, their fixations, and consequently their attentional focus, is displaced toward 30° eccentricity or further, creating a disadvantage for the central part of the visual field. The allocation of attentional resources has a limited capacity, which is even reduced in AD patients (Ballard et al., 2001). In case of high task demands, the participants have to employ most of their attentional resources to find the target. In this case, AD patients tend to allocate more resources toward the periphery, at the detriment of the resources normally engaged for central attention. The decline of visuo-spatial attention capacities in AD leads to a bias of the distribution of attentional resources, increasing attentional processing in the peripheral visual field.

As suggested by Green et al. (1993), if patients are aware of their problems in attending to visual information, especially in an early stage, they might compensate by attending more to the periphery and neglecting the center. This information is crucial for the rehabilitative approaches aiming at ameliorating visual attention deployment in space in AD patients.

Future studies should consider examining this question in a larger sample, including the question of head movements, and their potential role in the compensation for the deficits in attentional shifting. In the current study, the head of the participants was stabilized during the task. However, the present study offered the possibility to assess the patterns of visual exploration in AD patients in a large field of view, and to gain important information for the development of ecologically valid rehabilitative approaches for visual attention allocation impairments.

## Author Contributions

UM, RM, and TN contributed to the conception and organization of the research. LR, NG, and VV participated in the execution and data collection. VV, FF, and GZ designed the data analysis and statistical methods. VV executed the data analysis and statistical analysis. LR and DC contributed to the review and critique of the statistical analysis. VV wrote the first draft of the manuscript. All authors participated in the review and critique of the manuscript and approved the final manuscript.

## Conflict of Interest Statement

The authors declare that the research was conducted in the absence of any commercial or financial relationships that could be construed as a potential conflict of interest.
